# Myosin Light-Chain Kinase Is Necessary for Membrane Homeostasis in Cochlear Inner Hair Cells

**DOI:** 10.1371/journal.pone.0034894

**Published:** 2012-04-02

**Authors:** Guang-Jie Zhu, Fang Wang, Chen Chen, Lin Xu, Wen-Cheng Zhang, Chi Fan, Ya-Jing Peng, Jie Chen, Wei-Qi He, Shi-Ying Guo, Jian Zuo, Xia Gao, Min-Sheng Zhu

**Affiliations:** 1 MOE Key Laboratory for Model Animal and Diseases Studies, Nanjing Drum Tower Hospital and Model Animal Research Center of Nanjing University, Nanjing, China; 2 Zhejiang Provincial Key Lab for Technology & Application of Model Organisms, School of Life Sciences, Wenzhou Medical College, University Park, Wenzhou, China; 3 Department of Developmental Neurobiology, St. Jude Children's Research Hospital, Memphis, Tennessee, United States of America; University of Washington, Institute for Stem Cells and Regenerative Medicine, United States of America

## Abstract

The structural homeostasis of the cochlear hair cell membrane is critical for all aspects of sensory transduction, but the regulation of its maintenance is not well understood. In this report, we analyzed the cochlear hair cells of mice with specific deletion of myosin light chain kinase (MLCK) in inner hair cells. MLCK-deficient mice showed impaired hearing, with a 5- to 14-dB rise in the auditory brainstem response (ABR) thresholds to clicks and tones of different frequencies and a significant decrease in the amplitude of the ABR waves. The mutant inner hair cells produced several ball-like structures around the hair bundles *in vivo*, indicating impaired membrane stability. Inner hair cells isolated from the knockout mice consistently displayed less resistance to hypoosmotic solution and less membrane F-actin. Myosin light-chain phosphorylation was also reduced in the mutated inner hair cells. Our results suggest that MLCK is necessary for maintaining the membrane stability of inner hair cells.

## Introduction

Hearing is achieved through multiple steps; acoustic stimuli are received through the inner hair cells (IHCs) and outer hair cells (OHCs) in the organ of Corti, converted into electric signals, and then transmitted to the central nervous system [1]. These mechano-chemical and mechano-electrical transductions are performed by both cellular components and extracellular structures, and they involve many molecules in the inner ear, such as stretch-activated ion channels, caveolae, integrins, cadherins, growth factor receptors, myosin motors, cytoskeletal filaments, nuclei, extracellular matrix, and numerous other structures and signaling molecules [2]. The maintenance of membrane structure and the functional homeostasis of the cochlear cells are thus essential for these processes.

As critical sensors, hair cells function in signal transduction through apical hair bundles as well as somatic contractility and membrane machineries [2], [3], [4]. These cellular structures are important for the homeostasis of the cochlear hair cells and are highly regulated by cytoskeletal factors, including myosin motors, F-actin bundle proteins and F-actin crosslinking proteins [3], [5], [6]. Mutations in these proteins give rise to various types of hearing loss [7], [8], [9], [10]. It has been documented that several syndromic and non-syndromic types of hearing loss are caused by mutations of the myosin II genes, such as the p.R702H, p.R702C and p.R705H mutations in myosin heavy-chain 9 (MYH9) [11], [12], [13], [14] and the p.S7X, p.S120L, p.G376C and p.R726S mutations in MYH14 [15], [16]. Furthermore, recent reports have suggested that myosin II plays an important role in the development of the inner ear [17]. Therefore, similarly to other myosin motors, myosin II is thought to be important for hearing, but the underlying mechanism remains poorly understood. The activity of the myosin II Mg-ATPase may be regulated by myosin light-chain kinase (MLCK) through the phosphorylation of the myosin regulatory light-chain (RLC), leading to the modulation of multiple physiological processes [18]. We therefore speculated that MLCK might also play an important part in hearing.

MLCK has been implicated in regulating the contraction of smooth muscle and many important physiological processes, including cell migration, neurite growth, cytokinesis, cytoskeletal organization, platelet shape changes, secretion, and transepithelial permeability, through its phosphorylation of the myosin regulatory light-chain and through its non-kinase activities [18]. The smooth muscle *Mylk* locus has been shown to express three independent transcripts, including short *Mylk*, long *Mylk* and telokin, by alternative promoters [18], [19], [20]. Short MLCK has a catalytic core, a regulatory segment, three immunoglobulin (Ig)-like modules, a fibronectin module, a PEVK repeat-rich region, and a 3DFRXXL F-actin-binding motif [21], [22]. Long MLCK is identical to short MLCK but also contains a unique N-terminal extension of ∼900 residues. This extension region contains two Ig-like regions, four Ig-like regions and two DFRXXL motifs [23]. Several studies have suggested that the kinase activity of MLCK mediates important changes in cytoskeleton organization through RLC phosphorylation [24], [25], [26], [27], [28]. Several studies have also suggested that these non-kinase regions have versatile functions in cytoskeleton reorganization [23], [29]. In the present study, we found that hair cells expressed short MLCK, implying that MLCK might function in hearing. To investigate the function and regulatory mechanism of MLCK in hair cells, we analyzed an animal model with a specific deletion of MLCK in the IHCs and found that MLCK functioned in the membrane of these cells.

## Materials and Methods

All experiments were conducted in accordance with the guidelines set by the Animal Care and Use Committee of the Model Animal Research Center of Nanjing University (Nanjing, China) (permit number AP#MZ3).

### Generation of knockout mice with a deletion of *Mylk* in IHC

Floxed *Mylk* mice (*Mylk^flox/flox^*) in a congenic background (B6:129) were crossed with IHC*-Cre* transgenic mice [28], [30]. Because Cre driven by a prestin enhancer element is expressed in the IHCs beginning at postnatal day 14 [30], the resultant mice (*Mylk^flox/flox^:IHC-Cre*; or MLCK^IKO^) lacked MLCK in the IHCs. The littermates of the MLCK^IKO^ mice (*Mylk^flox/+^:IHC-Cre*) were used as controls (CTR). These mice were specific pathogen-free (SPF) and were maintained in standard animal rooms of the National Resource Center for Mutant Mice (NRCMM) of China.

### ABR recordings

Mice were anesthetized by intraperitoneal injection with Avertin at an initial dose of 500 mg/kg body weight, and the anesthesia was maintained with a half-dose delivered every 20 min. The ABR waveforms were recorded with subcutaneous needle electrodes at the vertex (active), the posterior bulla region of the right ear (reference), and the tip of the nose (ground) in a single-walled, sound-proof booth. An outlay trumpet was placed 3 cm in front of the nasal tip. Click and tone pips of 8, 16 and 32 kHz were generated using an evoked generation workstation system III (Tucker Davis Technologies Incorporated, Gainesville, FL, USA) powered by SigGen32 software. The response was averaged (n = 1024) and displayed from 110 dB to 0 dB, decreasing in 5 dB steps. The threshold was determined in each series of ABR waveforms as the lowest intensity that produced at least two clearly visible waves. All of the mice were allowed to recover after testing using a warm pad at 37°C.

### Western blot analyses

Western blot analyses were undertaken for the measurement of MLCK in the cochleae [28], [31], [32]. Briefly, the basilar membrane, including the hair cells, was isolated from postnatal mice (6–8 weeks). This tissue was frozen quickly in lysis buffer containing 20 mM Tris/HCl (pH 7.4), 50 mM NaCl, 1% Triton X-100, 1 mM phenylmethylsulfonyl fluoride and 10 mg/L aprotinin (Sigma–Aldrich, St Louis, MO, USA). After thorough homogenization, the sample was incubated on ice for an extra 30 min. The protein samples were prepared by boiling with loading buffer, followed by centrifugation. The protein concentration was measured using a bicinchoninic acid (BCA) protein assay (BioRad, Hercules, CA, USA). Equal amounts of protein were loaded for sodium dodecyl sulfate-polyacrylamide gel electrophoresis (SDS-PAGE), followed by protein transfer to a nitrocellulose membrane. The membrane was probed with a monoclonal antibody to MLCK (K36, Sigma–Aldrich, St Louis, MO, USA) followed by a secondary antibody. The MLCK signal was visualized by incubation with the Super Signal West Dura substrate (Pierce, Thermo, Rockford, USA) followed by exposure to film.

### Cochlear whole-mount *in situ* hybridization

The digoxigenin-labeled *Mylk* mRNA riboprobe (554 nt) used for the whole-mount *in situ* hybridization was prepared by *in-vitro* transcription from a cDNA fragment using a MAXIscript® In Vitro Transcription kit (Ambion, Applied Biosystems, TX, USA) according to the manufacturer's instructions, except that we synthesized the probe with 1.5 hours of extension at 37°C. The cDNA fragment cloned into the pBluescript KS+ plasmid vector (Stratagene, Agilent Technologies, CA, USA) was amplified from cDNA templates that were generated from mouse whole-embryo total RNA (*Mylk* mRNA accession number: BC058610) by reverse transcription using the following primers: EcoRI, forward, 5′-GAATTCGCAAGTGAAGCCAAAGACCA-3′; HindIII, reverse, 5′-AAGCTT CCTGCCCTTTCTTACAGTTC-3′. The mouse cochleae were fixed in 4% paraformaldehyde overnight at 4°C. They were dehydrated through a graded methanol series and stored at −20°C. Cochlear whole-mount *in situ* hybridization was conducted as described by Judice [33] with a modified hybridization buffer containing 50% (v/v) deionized formamide, 5× SSC20, yeast tRNA (0.3 mg/ml), heparin (0.1 mg/ml), CHAPS (1 mg/ml), EDTA (5 mM), Ficoll-400 (0.2 mg/ml), PVP (0.2 mg/ml), and BSA (0.2 mg/ml).

### Genotyping of isolated hair cells

Mouse IHCs and OHCs were isolated from the mouse cochleae based on a reported method for the isolation of gerbil IHCs [34]. The isolated hair cells were genotyped using a polymerase chain reaction (PCR) assay, after which the template DNA was extracted by the salting-out method. The PCR primer set for the left homologous arm was (a) 5′-TAGTGCGAGTGTCACTGTTG-3′; the primer set for the right homologous arm was (b) 5′-CCCCATGATTTGCCTCTAGT-3′.

### Histopathology

Mice were sacrificed with an overdose of anesthesia, and the acoustic capsule was removed. The cochleae were dissected out and infused with 4% paraformaldehyde in phosphate-buffered solution (PBS) through an open window over the cochlear apical turn. Decalcification was performed with 10% (W/V) ethylenediamine tetra-acetic acid (EDTA) for 5 days and followed by a standard histology examination. Briefly, the specimens were dehydrated in a graded series of ethanol solutions and subsequently embedded in paraffin. Sections were cut at 7-cm thickness and mounted onto positively charged slides. Standard hematoxylin and eosin (H&E) staining was conducted.

### Electron microscopy analyses

Mice were anesthetized with an overdose of Avertin. They were perfused with PBS followed by fixation with 2.5% glutaraldehyde in PBS. The cochleae were selected and post-fixed in 2.5% glutaraldehyde in PBS at 4°C for 4–6 h, followed by decalcification. For scanning electron microscopy (SEM), the surrounding bone, stria vascularis, and tectorial membrane were removed. These tissues were fixed in 1% osmium tetroxide, dehydrated, and critical point-dried. The samples were then mounted on stubs and sputter-coated with gold. Images were collected with an S-3000 N scanning electron microscope (Hitachi, Tokyo, Japan) at 15 kV. For transmission electron microscopy (TEM), the samples were post-fixed in 1% osmium tetroxide, dehydrated, infiltrated, and polymerized in araldite. Ultrathin sections were post-stained and examined using a Hitachi-7650 transmission electron microscope (Hitachi Software Engineering, Yokohama, Japan).

### Hypoosmotic assay

IHCs were isolated from mouse cochleae based on a reported method for the isolation of gerbil IHCs [34]. An accessory pipette (diameter, 50–60 µm) fabricated from a 3-mm thin-walled glass tube was used to manipulate the IHCs. The isolated IHCs were transferred into 20 µL isosmotic D-Hank's solution containing NaCl (136.9 mM), KCl (5.4 mM), Na_2_HPO_4_ (0.3 mM), NaHCO_3_ (4.2 mM), and KH_2_PO_4_ (0.4 mM) at pH 7.2, adjusted to 300 mOsm. The cell shape in isosmotic solution was imaged using a Leica confocal laser scanning microscope (Leica, HD, GER). Subsequently, 40 µl of distilled water was carefully added onto the edge of the water bead without moving the cell. Cell images were obtained 10 min after this hypoosmotic treatment. The cell size was analyzed with the ImageJ software (http://imagejdev.org/downloads). The cell volume was calculated using the following formula: V = 4/3πr^3^ (V: volume; r: radius of the cell).

### Immunofluorescence assay for cross-sections of the Corti's organ and inner ear sensory epithelia

To isolate the basilar membrane, the cochleae were removed and infused with 4% paraformaldehyde in PBS through the round window followed by post-fixation with the same buffer at 4°C for 3 h. After decalcification in 10% EDTA at room temperature overnight, the Corti sensory epithelia was dissected from the soft cochlea. Then, the cochleae were dissected, fixed in 4% paraformaldehyde overnight, decalcified with 10% EDTA at room temperature for 5 days, dehydrated through an ethanol gradient, embedded in paraffin and sectioned. The immunohistochemical analysis of the sections was performed after antigen retrieval by autoclaving. The sensory epithelia and sections were permeabilized with 0.5% Triton X-100 in PBS for 20 min and washed three times with PBS. Nonspecific binding was blocked by incubation with PBS containing 5% goat serum, 1% BSA, and 0.1% Triton X-100 for 1 h at room temperature. The tissues were incubated with primary antibody overnight at 4°C and washed three times with 0.1% Tween in PBS, followed by incubation with secondary antibody for 1.5 hours at room temperature. Anti-MLCK (K36, 1∶200, Sigma–Aldrich, St Louis, MO, USA), anti-phospho myosin light-chain 2 (Ser 19) (1∶150 dilution; Cell Signaling, Cell Signaling Technology, BSN, USA) and anti-myosin VII (1∶200 dilution, Santa Cruz Biotechnology, CA, USA) were used as primary antibodies. The specificity of the K36 anti-MLCK antibody for immunofluorescence analysis was tested in MLCK-deficient smooth muscle, as we previously reported [28], [31], [35]. The smooth muscles from *Mylk^flox/flox^*; SM22CreERT2 mice that received tamoxifen treatment did not stain with MLCK, whereas a clear MLCK signal was observed in control airway [31], intestinal [28] and vascular smooth muscles [35]. Reports from other labs also suggest that the K36 antibody works well for immunostaining [36], [37]. Alexa Fluor 488-conjugated goat anti-rabbit IgG and Alexa Fluor 555-conjugated goat anti-mouse or anti-rabbit IgG were used as the secondary antibodies (Molecular Probes, Carlsbad, CA, USA). The specificities of the secondary antibodies were tested by comparison experiments with or without primary antibodies. F-actin was labeled by Alexa Fluor 488-conjugated phalloidin (Invitrogen, CA, USA). Topro dye (Invitrogen, CA, USA) was used to stain the nucleus. The immunofluorescence signals were examined under a Leica confocal microscope.

### Data analysis

The data are represented as the means ± S.E. Significant differences between groups were determined by Student's *t*-test.

## Results

### MLCK expression in the cochleae

**Figure 1:**
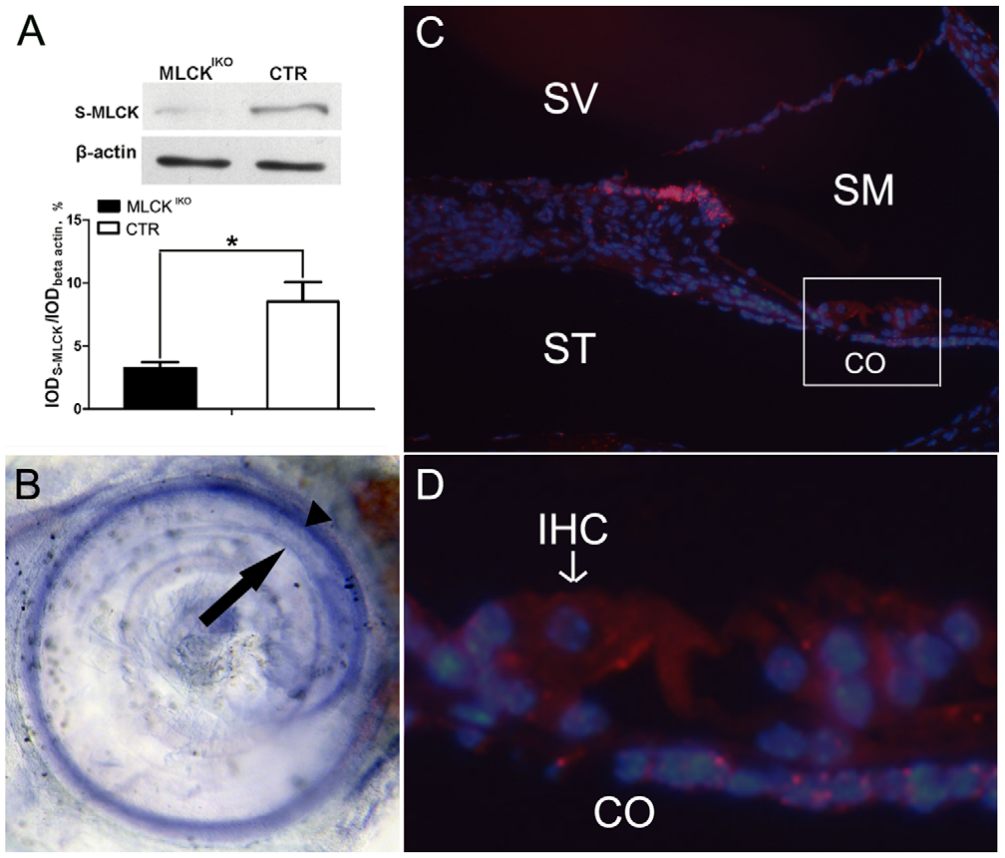
MLCK expression in mouse cochleae. (A) Upper panel: Western blot analyses demonstrate that S-MLCK (short MLCK) is expressed in mouse cochleae. Samples of 25 µg of cochlear protein were separated on 5% SDS-PAGE. The K36 antibody was used as the primary antibody. The results showed one band of approximately 130–140 kD (short MLCK should be 108 kD). Lower panel: Quantitative analysis of *Mylk* gene expression in MLCK^IKO^ (solid symbol) and CTR (open symbol) mice. The bars represent the means of IOD_S-MLCK_/IOD_βactin_ ±SD (integral optical density, IOD). The * symbols denote statistical significance at the level of P<0.05 *vs* CTR. (B) Whole-mount *in situ* hybridization for *Mylk* RNA in the inner ear. *Mylk* mRNA is expressed predominantly in the stria vascularis (arrowhead) and is moderately expressed in the organ of Corti (arrow). (C) Immunochemistry of K36 (red) in cross-sections of cochleae from wild-type mice. The nuclei were stained (blue) with Topro. (D) High-magnification view of the organ of Corti. S-MLCK: short MLCK; CO, the organ of Corti; SV, scala vestibuli; SM, scala media; ST, scala tympani; IHC, inner hair cell.

MLCK is believed to be expressed ubiquitously, but its expression profile in the inner ear has not been documented. To measure MLCK expression in the inner ear, we conducted western blotting of the MLCK protein and *in situ* hybridization of the *Mylk* mRNA. The 108-kD MLCK protein (short MLCK) was detected clearly in the cochlear basilar membrane, but the 220-kD MLCK isoform (long MLCK) was not. Compared with CTR mice, MLCK^IKO^ mice showed a lower expression of S-MLCK (3.93±0.50 *vs* 8.51±1.56, P<0.05, n = 3) [Fig. 1A]. Whole-mount *in situ* hybridization for the organ of Corti showed that the *Mylk* mRNA was found predominantly in the vascular plexus and was moderately expressed in the inner ear sensory epithelia [Fig. 1B]. Immunohistochemistry was conducted to localize the MLCK protein in the hair cells. Moderately strong signals were observed in the IHCs and OHCs [Fig. 1, C and D]. Positive MLCK signals were also found in other cells around the epithelia. This result showed that MLCK was expressed in hair cells and suggested a potential function in these cells.

### Targeted disruption of MLCK in IHCs

**Figure 2:**
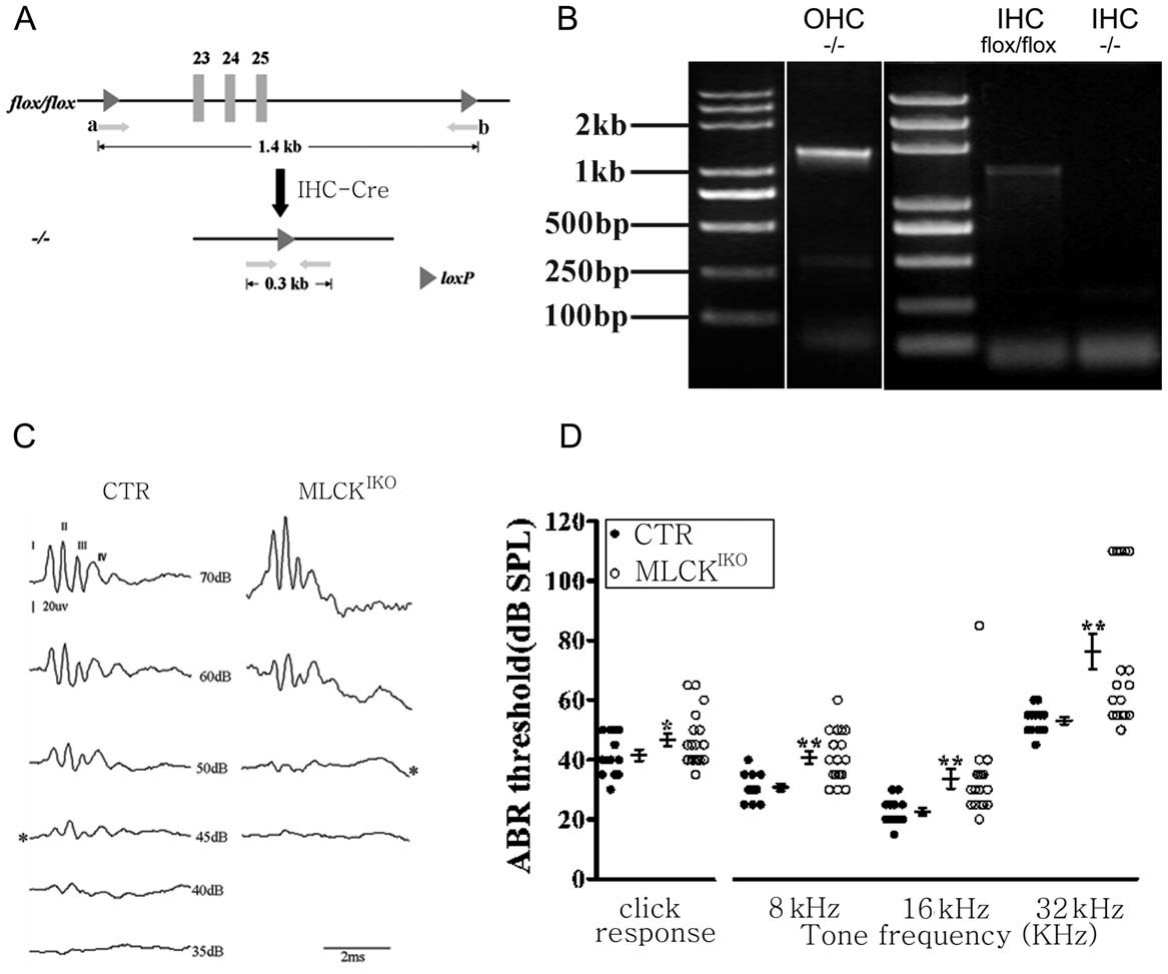
Disruption of the *Mylk* gene in IHCs and ABR threshold analyses. (A) Generation of knockout mice with deletion of *Mylk* in the IHCs (schematic). The 1.4-kb genomic DNA fragment contains *Mylk* exons 23–25 (*Mylk* accession number: NC_000082), which encode the adenosine triphosphate-binding site of the kinase. Mice containing the floxed allele were crossed with IHC-Cre mice to generate *Mylk^flox/+^* (CTR) and *Mylk^flox/flox^;IHC-Cre* (MLCK^IKO^) mice. The locations of PCR primers *a* and *b* are indicated by arrows. (B) DNA isolated from CTR and MLCK^IKO^ mice IHCs and OHCs was analyzed by PCR; the 1.4-kb and 308-bp products represent CTR and mutated MLCK, respectively. (C) Representative ABR waveforms measured in CTR and MLCK^IKO^ knockout mice at 4–6 months in age measured in response to click stimuli of the indicated intensities. *ABR threshold. (D) Summary of the ABR thresholds for click and tone stimuli in 4- to 6-month-old control (filled circles) (n = 13) and MLCK-null mice (open circles) (n = 18). Averaged data (means ± S.E.) are shown for the click and tone stimuli. *Significant difference compared with control mice are shown. *, *P*<0.05; **, *P*<0.01.

To delete MLCK in the inner hair cells, mice with floxed *Mylk* (*Mylk^flox^* allele) were crossed with IHC-Cre mice expressing a Cre recombinase driven by a regulatory element of the *prestin* gene [30] [Fig. 2A]. To confirm the tissue-specific ablation of MLCK, we separated and collected individual hair cells and confirmed the deletion of exon 23–25 through a single-cell PCR analysis. The IHCs from MLCK^IKO^ mice produced the expected 0.3-kb fragment (with deletion of exons 23–25), whereas the OHCs produced a strong 1.4-kb fragment (without the deletion of exons 23–25) and a weak 0.3-kb fragment. In contrast, wild-type IHCs produced only the 1.4-kb fragment. The identities of the 0.3 kb and 1.4 kb were confirmed by sequencing. Thus, this result indicated that MLCK was successfully deleted in IHCs [Fig. 2B]. Western blotting of the basilar membrane also supported this conclusion [Fig. 1A]. However, a minor deletion also occurred in the OHCs. Such non-specific leakage is unlikely to have affected the IHC phenotype because no phenotype was observed upon the deletion of MLCK in the OHCs (our unpublished data).

### MLCK-deficient mice display an elevation of the hearing threshold

**Figure 3:**
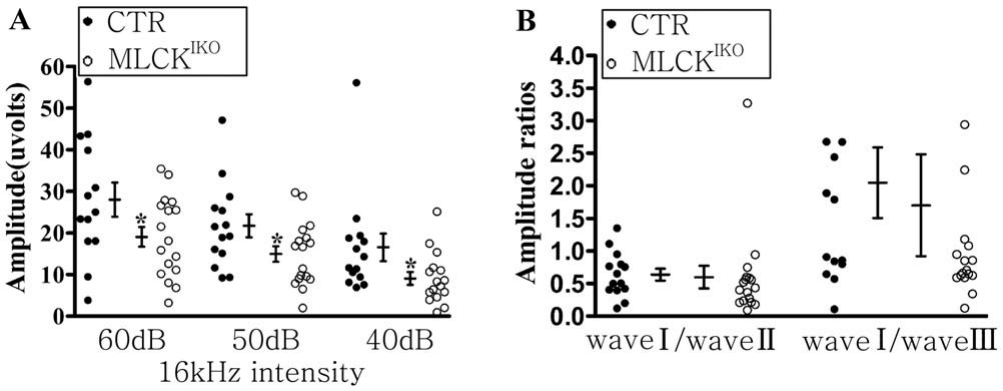
ABR waveform analyses in CTR and MLCK^IKO^ mice. (A) The ABR amplitudes of wave I in wild-type mice (filled circles) and MLCK^IKO^ mice (open circles) at the indicated click intensities. Averaged data (means ± SEM) are shown for tone (16-kHz) stimuli, n = 13 and 18 for wild-type and MLCK^IKO^ mice, respectively. *Significant difference compared with wild-type mice are shown. *, *P*<0.05. (B) The amplitude ratios of ABR wave I/wave II and wave I/wave III shown for each mouse. No significant difference was observed.

Control (CTR) and MLCK^IKO^ mice (age, 4–6 months) were subjected to ABR waveform measurement. The ABR waveforms in response to clicks or tones at different intensities contained at least four distinct peaks (waves I–IV) [Fig. 2C]. These peaks corresponded to cochlear activity (wave I) and neural activity (waves II–IV). Hearing function was evaluated based on the ABR thresholds, frequency dependency, and wave amplitude. Compared with CTR mice, MLCK^IKO^ mice displayed a significantly higher threshold in response to clicks (46.67±9.07 dB *vs* 41.50±6.89 dB in CTR, *P*<0.05) and tones (8 kHz: 40.83±8.62 dB *vs* 30.77±4.49 dB in CTR, *P*<0.01; 16 kHz: 33.61±14.02 dB *vs* 22.69±4.39 dB in CTR, *P*<0.01; and 32 kHz: 76.39±25.02 dB *vs* 53.08±4.35 dB in CTR, *P*<0.01) [Fig. 2D]. In light of the hypothesis that the sensitivity to different frequencies of sound varies with the region of the hearing epithelia [38], this result implied that the IHCs in a wide area were affected. Further analyses of the waveforms showed that the wave-I amplitudes on 60 dB, 50 dB and 40 dB in response to a tone (16 kHz) decreased by 7.75 µV on average, but the wave I/wave II ratio did not change (p>0.05) [Fig. 3]. This result suggested impairment in the proximal inner ear but no effect on central regulation. This featured pathology implies that such a phenotype might be unlikely to be attributed to the non-specific expression of Cre in the cerebellum, testis and other tissues of IHC-Cre mice [30].

### The deletion of MLCK led to the deformation of IHCs through the generation of a ball-like structure

**Figure 4:**
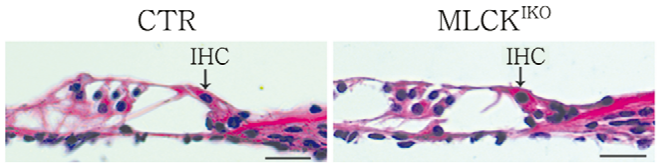
Hematoxylin & eosin (H&E)-stained sections of the organ of Corti in CTR and MLCK^IKO^ mice. Arrows indicate inner hair cells. The structure of the organ of Corti appears normal in 4-month-old MLCK^IKO^ mice (n = 5). Scale bars: 50 µm.

**Figure 5:**
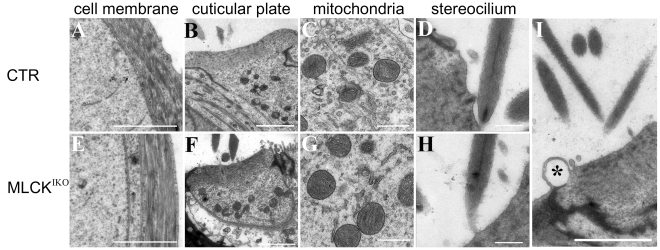
Transmission electron microscopy analyses of IHC microstructure. IHCs from 4-month-old CTR (A–D) and MLCK^IKO^ (E–H) mice were subjected to ultrastructural analysis. “Bubbles” (indicated by asterisks) are located at the top of the cuticular plate in the mutant IHCs (I). The structures of the membranes of the IHC lateral wall (E), cuticular plate (F), mitochondria (G) and stereocilium (H) in IHCs are similar in mice of both genotypes. Scale bars: 2 µm (A, B, E, F and I) or 500 nm (C, D, G and H).

**Figure 6:**
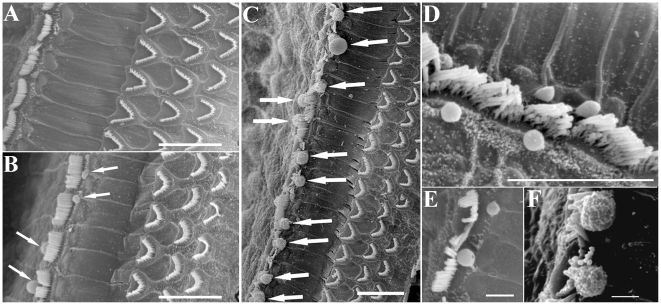
Scanning electron microscopy analyses of CTR (A) and MLCK^IKO^ (B–F) mice. (A) CTR mice show normal hair bundles in one row of IHCs and three rows of OHCs on the basilar membrane (BM). (B–F) In MLCK^IKO^ mice, several ball-like structures were observed around the hair bundles of the IHCs (arrows). Scale bars: 10 µm (A–D) or 2 µm (E–F).

To examine the histological structure of the organ of Corti, cochlear slides were stained with H&E and observed under light microscopy. In the knockout mice, the cochlear histology was normal with respect to the morphologies of the IHCs, OHCs, Hensen's cells and other cells around the organ of Corti [Fig. 4]. TEM analyses showed that the mutant IHCs had normal nuclei, cellular organelles and tight junctions. A careful examination of the cuticular plate and hair stereocilia showed a normal morphology and electronic density in these structures. Interestingly, several of the mutant IHCs produced “bubbles” at the top of the cuticular plate and in connection with the cytoplasm and membrane structure [Fig. 5]. SEM examination revealed ball-like structures around the IHC stereocilia in approximately 85% of the mutant IHCs [Fig. 6]. The IHCs from younger (<6 weeks) knockout mice showed a lower formation frequency (<30%). The diameters of these ball-like structures ranged from 1.21 µm to 3.76 µm (mean, 1.97 µm). The ball-like structures were not observed in CTR cochleae. Our results suggested that these ball-like structures were produced by the mutated inner hair cells.

### MLCK-deficient IHCs were sensitive to hypoosmotic treatment

**Figure 7:**
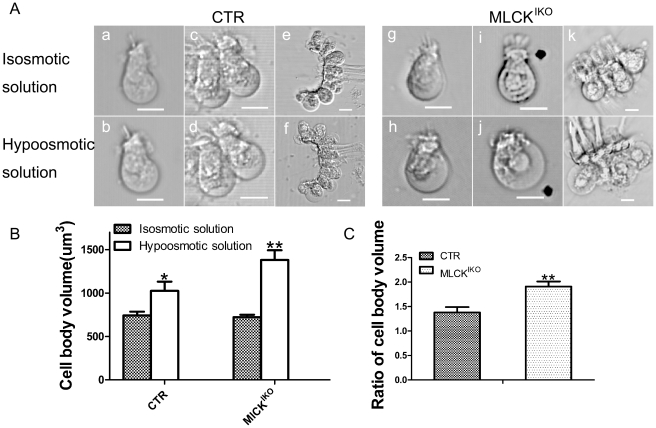
Volume alteration of isolated IHCs upon hypoosmotic treatment. (A) The morphology of IHCs isolated from 4-month-old CTR (a, c, e) and MLCK^IKO^ mice (g, i, k) in isosmotic D-Hank's solution. CTR (b, d, f) and MLCK^IKO^ (h, j, l) IHCs were also imaged after treatment with hypoosmotic solution (a mixture of D-Hank's solution and water at a ratio of 1∶2) for 10 min. Scale bars: 10 µm. (B) Quantification of the change in IHC volume induced by hypoosmotic treatment. (C) The ratio of the changes in the cell body volume induced by hypoosmotic treatment. The error bars represent means ± S.E., *P<0.05, **P<0.01.

**Figure 8:**
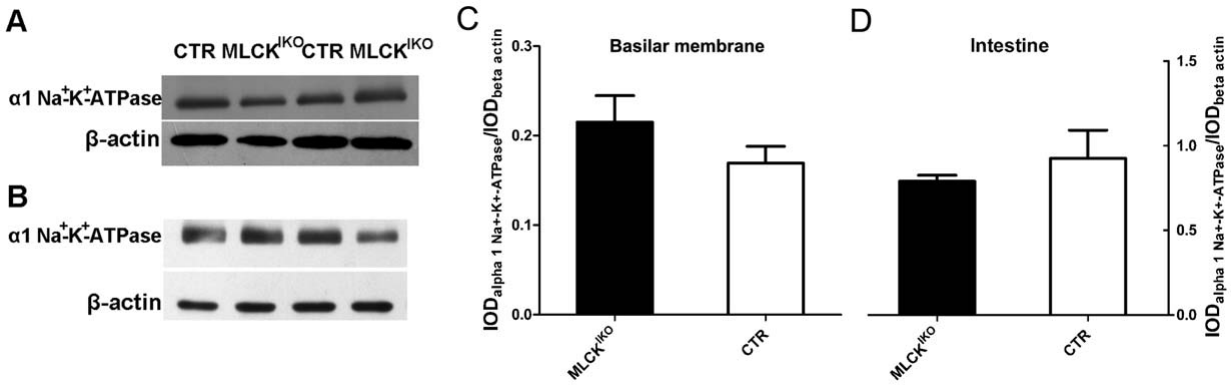
Expression of the Na^+^-K^+^-ATPase protein. (A, C) The apical turns of the basilar membrane were isolated from 10-week-old MLCK^IKO^ and CTR mice and subjected to western blotting to analyze Na^+^-K^+^-ATPase protein expression. (B, D) Smooth muscle from ileum segments of *Mlck* ^SMKO^ mice (28) was isolated and subjected to western blotting to analyze Na^+^-K^+^-ATPase protein expression. Quantitative analysis was performed by measuring the relative density of the bands. The bars represent the means of IOD_α1 Na + - K+-ATPase_/IOD_βactin_ ± S.E. (Fig B: P>0.05 *vs* CTR; Fig D: P>0.05 *vs* CTR).

Changes in the volume of hair cells is important for hearing sensitivity [39], [40], and the cell volume is primarily regulated by membrane tethering and cytoskeleton organization [41]. The results of our morphological analyses led us to speculate that the ball-like structures might be formed by an altered membrane tether force. To test this hypothesis, we treated isolated fresh IHCs with 30% physiologic solution (0.3% NaCl). After treatment, the volume of the IHCs from mutant mice increased significantly, from 722±28 µm^3^ (n = 4) to 1381±112 µm^3^ (n = 4) (*P*<0.01); the volume of the CTR IHCs also increased significantly, from 742±44 µm^3^ (n = 4) to 1024±107 µm^3^ (n = 4) (P<0.05). However, the relative increase in the volume of the MLCK-deficient IHCs was significantly higher than that of the CTR IHCs (1.91±0.10 *vs* 1.38±0.11, P<0.01). To verify this effect in the mutant IHCs, we conducted the same experiment using hypoosmotic glucose buffer. A similar result was obtained (data not shown). To exclude the possibility of injury to the IHC membrane from mechanical separation, we subjected an IHC cell mass to hypoosmotic treatment, and similar results were obtained [Fig. 7]. This finding suggested that the deletion of MLCK caused increased sensitivity to hypoosmotic solution, and the MLCK-deficient IHCs showed a low membrane tension compared with control samples. To rule out the possible effects of alteration of the ion channels responsible for hypoosmotic treatment after MLCK deletion, we measured the expression of the Na^+^-K^+^-ATPase gene, an important pump for fluid homeostasis in the inner hair cells. The Na^+^-K^+^ -ATPase protein level in either the mutant cochlear basilar membrane or MLCK-deficient smooth muscle tissue was comparable to that in control tissues [Fig. 8]. Therefore, our results suggested that the deletion of MLCK did not affect Na^+^-K^+^-ATPase expression.

### MLCK-deficient IHCs show reduced F-actin and RLC phosphorylation

**Figure 9:**
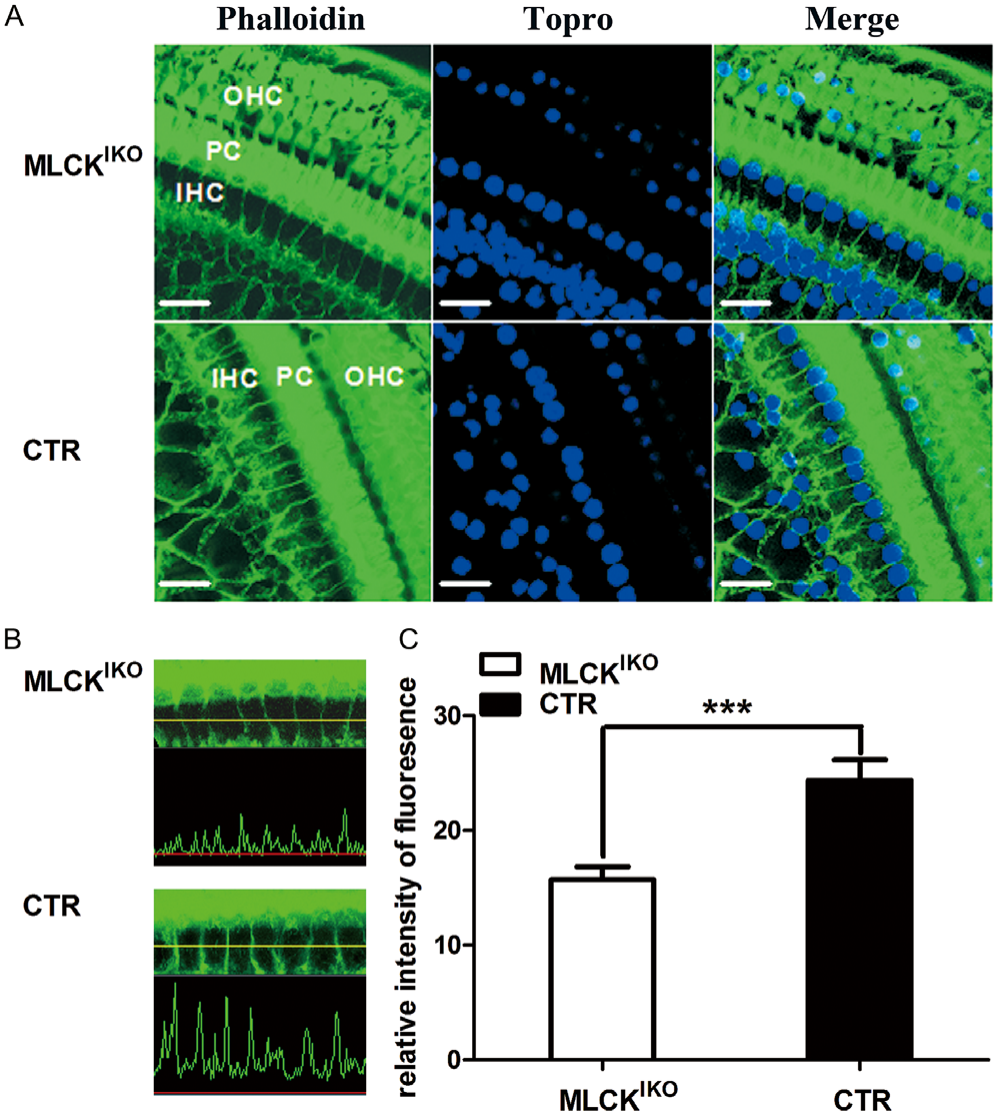
The actin cytoskeleton in the basilar epithelium of the organ of Corti. (A) In the cochleae of MLCK^IKO^ mice, staining with phalloidin reveals decreased F-actin formation in the cytoplasm and cell membrane of IHCs, whereas its expression in pillar cells (PCs) and OHCs remains strong in comparison with that of CTR mice. The nuclei were stained (blue) with Topro. Scale bars: 20 µm. (B) Representative waveforms of F-actin fluorescence signals in CTR and MLCK^IKO^ inner hair cells, captured by a LOYMUPS FLUOVIEW Ver.1.7.a Viewer. (C) Quantification of the relative intensity of the F-actin fluorescence signals in CTR (solid symbol, n = 10) and MLCK^IKO^ inner hair cells (open symbol, n = 13). The bars represent the means ± S.E., *Significant difference compared with CTR. ***P<0.001.

The enriched F-actin mesh structure underneath the lipid layer is critical for the tethering tension or pliability of the cell membrane. To determine whether the F-actin density was altered in the MLCK^IKO^, we stained the inner ear cells with phalloidin. Most of the phalloidin signal was distributed around the IHC cell membrane and the button area. The CTR IHC had strong and continuous F-actin staining along its membrane structure. However, the F-actin staining in MLCK-deficient IHCs was weak and discontinuous [Fig. 9A]. Quantitation of the relative F-actin density underneath the membrane at the lumbus of the cell showed that the mutant IHC had 64.43% of the density of the control (15.7±1.12 *vs* 24.40±1.76, p<0.001) [Fig. 9, B and C]. The IHCs and OHCs displayed similar staining of myosin VII, a hair cell-specific structural protein in mice [Fig. 9A]. This result indicated that the polymerized F-actin underneath the IHC membrane was reduced after MLCK deletion.

**Figure 10:**
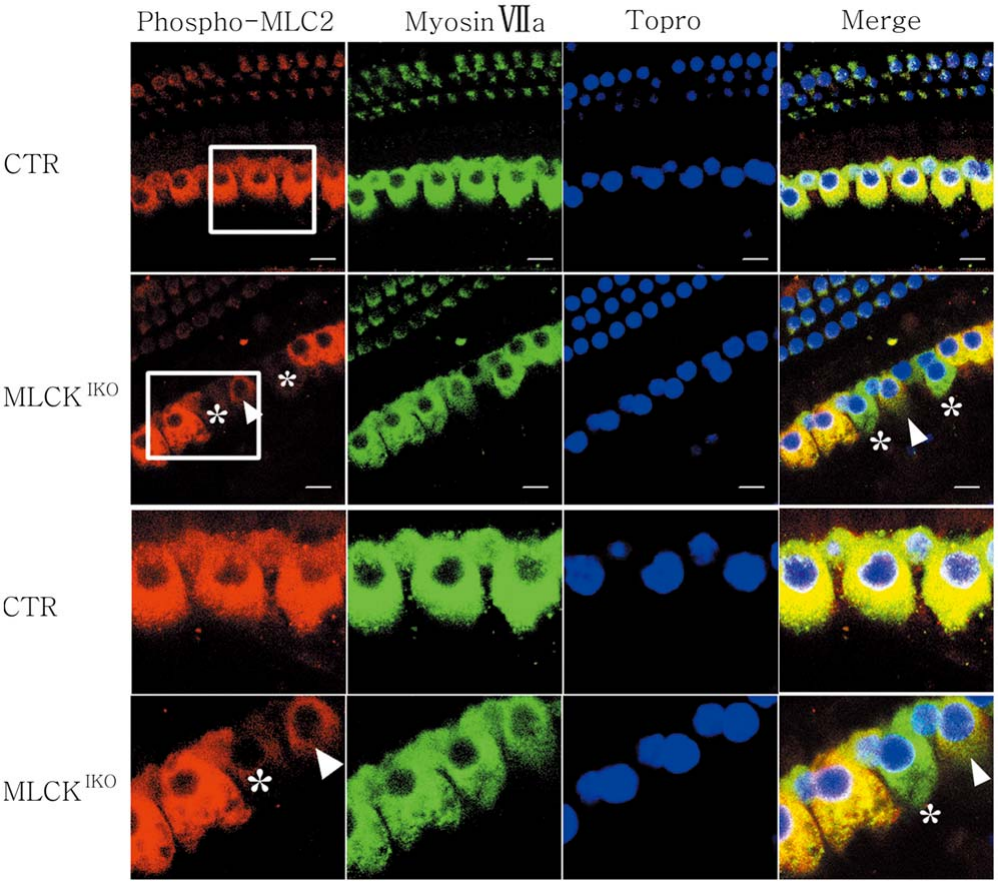
Immunohistochemistry of phospho-MLC2 and myosin VIIa in the basilar epithelium. In MLCK^IKO^ mouse cochleae, the phospho-MLC2 signal (red) (asterisks and arrowhead) in 60–70% of IHCs was weaker than that of the CTR IHCs, whereas myosin VIIa staining (green) was similar in both CTR and MLCK^IKO^ IHCs. The nuclei were stained (blue) with Topro. Scale bars: 10 µm.

MLCK is a dedicated kinase for myosin light-chain phosphorylation. In addition to its central role in smooth-muscle contraction [18], [28], RLC phosphorylation is also involved in various cell processes [18]. There is evidence that the phosphorylation of the myosin light-chain enhances the formation of polymerized F-actin [42]. We therefore measured RLC phosphorylation in IHCs by staining for phosphorylated RLC with a specific antibody and quantifying its relative staining density over myosin VII with a modified ImageJ software [43]. In MLCK^IKO^ cochleae, many IHCs showed obviously weak staining of phosphorylated RLC in contrast to CTR [Fig. 10]. The average value for phosphorylated RLC was significantly lower than that of CTR (92.11±58.72 *vs* 169.33±20.92, p<0.01). However, approximately 30–40% of the mutant inner hair cells exhibited phosphorylated RLC staining similar to that observed in the CTR cells. This observation might be due to the variation in knockout efficiency in individual IHCs [Fig. 10].

## Discussion

Hearing loss is one of the most common sensory deficits in humans, and >50% of congenital cases of hearing loss are caused by genetic factors [44], [45], [46]. Linkage analyses have been used to identify the genetic correlations between genes and diseases [47]. However, investigations of hearing loss are limited because the inner ear is positioned deep within the temporal bones, and the physical examination of the human inner ear is possible only *post mortem*. Thus, animal models are very useful for mechanistic studies. Moreover, hearing is a complex process that is coordinated by different cells in the organ of Corti, the neuronal system and other systems (e.g., the vascular system). Assessing the function of a ubiquitously expressed gene exclusively in IHCs is therefore difficult. To circumvent these difficulties, we specifically deleted the *Mylk* gene in inner hair cells by crossing floxed *Mylk* mice with transgenic mice that express Cre in the IHCs. Mice with successful ablation of *Mylk* are a useful model for elucidating MLCK function in IHCs.

Several cytoskeletal proteins, such as actin bundling proteins, non-conventional myosin molecules, cadherins, and Rho small GTPases, have been implicated in the function of hair cells [7], [9], [10], [11], [48], [49], [50], [51]. Mutations in these molecules cause structural abnormalities of the hair bundles, the cuticular plate, and stereocilial cross-linkage, resulting in the impairment of acoustic transduction [7], [9], [10], [11], [49]. MLCK is a potent regulator of cytoskeletal organization, so it is expected that this protein could play a critical role in the formation of these hearing structures. However, this study showed that MLCK deletion did not affect these structures in IHCs. This finding suggests that MLCK is not necessary for the formation of the stereocilia or the cuticular plate. However, the deletion of MLCK caused IHC membrane deformation, as indicated by the formation of ball-like structures, altered resistance to hypoosmotic treatment and reduced membrane F-actin staining. We thus suggest that MLCK might regulate the tether force of the IHC membrane, possibly through F-actin network formation, thereby regulating IHC membrane stability and proximal hearing transduction. This finding revealed a novel role for MLCK in acoustic transduction.

In OHCs, membrane homeostasis has important functions in hearing amplification. The underlying mechanism may involve ion channels, the membrane tether force and other membrane-associated events [51], [52]. Thus, hearing impairment due to MLCK deletion in IHCs may be caused by multiple factors. Among these possible factors, the formation of the ball-like structures may affect the rheology of the endolymphic fluid and hence inhibit the transduction of vibrations to the IHC hair bundles through this fluid [53].

Based on the biochemical properties of MLCK, the regulation of membrane tension by MLCK might occur through several mechanisms. First, MLCK may strengthen the cell membrane through its non-kinase activities. The non-catalytic N-terminal extension of MLCK can bundle F-actin; it can also bind to myosin and interact with membrane proteins, thereby enhancing the cytoskeletal mesh structure underneath the lipid layers of the membrane [21], [22], [23], [54]. However, we did not detect the expression of long MLCK in IHCs. We therefore suggest that the N-terminal extension of MLCK might not be involved in the regulation of membrane tension. Second, MLCK might strengthen the cell membrane through its kinase activity. Increased myosin light-chain phosphorylation caused by the constitutive expression of active MLCK is correlated with increased cytoskeletal stiffness and reduced cell volume in fibroblasts *in vitro* [55]. As described in the present study, the deletion of MLCK in IHC cells led to decreased myosin light-chain phosphorylation and reduced amounts of F-actin throughout cells. This finding suggested that MLCK might regulate membrane F-actin through an RLC phosphorylation-dependent mechanism. Short MLCK also contains three repeat motifs (DFRXXL) in its N-terminus, but it is unlikely that this protein has a critical function in the context of F-actin bundling because its bundling activity is much weaker [23].
